# Age-Tastic: An Evidence-Based Intervention to Improve Health, Safety, and Well-Being in Older Adults

**DOI:** 10.1093/geroni/igab046.1910

**Published:** 2021-12-17

**Authors:** Manoj Pardasani, Jacquelin Berman, Mebane Powell, Madison Gates

**Affiliations:** 1 Adelphi University, Garden City, New York, United States; 2 NYC Department for the Aging, New York, New York, United States

## Abstract

Age-Tastic! is a holistic intervention that enhances the well-being, health and safety of older adults. Most evidence-based interventions aimed at older adults have focused on singular aspects of health such as cognitive health, falls prevention, depression, advanced care planning, etc. There are few interventions that encompass a holistic approach to health and safety. Age-tastic! is one such intervention that encompasses various aspects of health – social support, financial well-being, physical safety, mental health, health care management, and nutrition. Designed as a competitive board game to entice older adults, this intervention integrates concepts of cognitive restructuring, behavioral activation and game theory to educate, motivate and encourage healthful behaviors. At the core of this intervention is a focus on increasing awareness of health and safety issues, improving health literacy and changing harmful behaviors. A randomized control trial was conducted with 98 older adults assigned to an experimental and control group. Interviews were conducted at baseline, right after the intervention ended (8 weeks) and again after a short time (8 weeks after intervention ended). The results showed significant increases among experimental group participants in knowledge of healthy behaviors (p=0.05), perception of self-efficacy for engaging in healthful behaviors (p<0.001) and engagement in health behaviors (p=0.001). Regression analyses demonstrated that greater knowledge and self-efficacy were associated with behavioral change within the intervention group (p<0.001). Knowledge about the importance of preventing falls and communication with medical providers was positively correlated with the corresponding behavioral change (p<0.05). Implications for health literacy among older adults will be shared.

